# Testing of how and why the *Terpios hoshinota* sponge kills stony corals

**DOI:** 10.1038/s41598-021-87350-4

**Published:** 2021-04-07

**Authors:** Siang-Tai Syue, Chia-Hsuan Hsu, Keryea Soong

**Affiliations:** Department of Oceanography, National Sun Yat-Sen University, Kaohsiung, 80424 Taiwan

**Keywords:** Ecology, Biodiversity, Tropical ecology

## Abstract

An encrusting sponge, *Terpios hoshinota,* has the potential to infect all species of stony corals in shallow reefs and killing them. It caused a decline in coral coverage in two south-eastern islands of Taiwan. We proposed two hypotheses to examine how the sponges kill the corals, namely, light blocking and toxins, and tested by in-situ experiments. The results revealed that both light blocking, sponge toxins, and particularly the combination of both factors were effective at inducing tissue damage in stony corals over a short period. Second, to answer why the sponges killed the corals, we tested two hypotheses, namely, gaining nutrients versus gaining substrates for the sponge. By analyzing the stable isotopes ^13^C and ^15^N, as well as exploiting an enrichment experiment, it was possible to determine that only approximately 9.5% of the carbon and 16.9% of the nitrogen in the newly grown sponge tissues originated from the enriched corals underneath. The analysis also revealed that the control corals without isotope enrichment had higher δ^13^C and δ^15^N than the control sponges, which was an additional indication that *T*. *hoshinota* did not rely heavily on corals for nutrients. Therefore, our results support the hypothesis that the encrusting sponge did not kill corals for food or nutrients, but rather for the substrate.

## Introduction

Coral reefs are being threatened by global warming and ocean acidification^1^. In addition, coral diseases and predators have been reported to cause mortality and decline in coral coverage in reefs worldwide^2,3^. It remains unclear whether these adverse biological factors are also related to global change, and whether their magnitudes and frequencies may significantly contribute to the decline of coral reefs before the physical and chemical effects become manifest^4^.

Sponges are organism that often interacts with reef corals; the two groups could interact in the following scenarios: competition when both parties suffer (−, −); predation or parasitism, whereby one suffers but the other benefits (−, +); or mutualism, whereby both parties benefit (+ , +)^5^. Both groups are sessile, and therefore require a substrate, which is a critical resource in reef habitats^6^. Both are often symbiotic with photosynthetic microbese.g.,^7,8^ indicating that they may depend on and compete for light as well as energy sources. Their relationship may be critical to the reef environment when one or both parties become abundant because the dynamics and the future of reefs depends, at least partially, on the relationship.

In 2006, a population explosion of an encrusting, black sponge *Terpios hoshinota* was first reported in a reef survey of the fringing reefs of Green Island and Orchid Island to the east of Taiwan in the western Pacific; most of the substrate the sponge covered were once live stony corals^9^. The species also caused substantial coral mortality in Guam^10^, Ryukyu Islands^11^, and Yongxing Island^12^ and Taiping Island^13^ in the South China Sea, and has been recorded in a growing number of areas, including American Somoa, Truk Lagoon in Micronesia, Cebu Island in the Philippines, North Mariana Islands, Ryukyu Archipelago, Thailand, Indonesia, Maldives, Mauritius, Palk Bay, and Lizard Island in the Great Barrier Reef^10,11,14–21^.

Despite having several defense mechanisms against benthic neighbors, stony corals appear defenseless when encountering *T. hoshinota*^9,11^. Nevertheless, it remains unclear how the sponge kills corals because several types of interaction have been observedsee^22^. Bryan^10^ suggested toxins as a possible cause because it was observed that small sponge fragments induce the retraction of the coral polyps, as well as lead to coral tissue death^10^. This hypothesis was supported by the discovery of secondary metabolites isolated from *T. hoshinota*, such as nakiterpiosin and nakiterpiosinone, which have been reported to have the ability to kill the mouse lymphocytic leukemia cell, P388, in laboratories e.g.,^23^. However, whether the toxin is released by *T. hoshinota* when interacting with stony corals remains inconclusive.

The second possibility of the killing mechanism is potentially related to the unusual dark color of *T. hoshinota* see^24^. Some zooxanthellate corals are known to release ammonia, a nutrient in oligotrophic waters of most coral reef environments, in the dark^25^. Therefore, it is possible that the black color of *T. hoshinota* first evolved as a mechanism to induce nutrient releasing of corals but resulted in killing corals due to long periods of coverage. Experiments that distinguish darkness from toxins could potentially reveal how the sponge attacks the corals.

In addition to proximate factors, such as the killing mechanism, the ultimate factors, i.e., the benefits gained by the sponge from killing corals, could also be investigated. Bryan^10^ reported that *T. hoshinota* grew faster on the massive coral, *Porites lutea,* than on reef substrate^10^. Lin and Soong^16^ reported a similar finding after comparing the expansion rates of the sponges on live corals versus those on other substrates^16^. The underlying mechanisms could be the nutrients available in coral tissues, although other possibilities, such as the substrate provided by corals, could also be the real incentive for killing the corals. By contrast, Plucer-Rosario^14^ investigated the growth rates of *T. hoshinota* on branches of *Acropora formosa*, comparing live segment controls with segments cleared of tissues^14^. The results indicated that the sponge grew faster on the latter, which is incompatible with the nutrient hypothesis. Experiments that focus on various factors (not only growth or expansion rates) could further test the ultimate causes, or the adaptive value of killing corals.

Two questions were explored in this study, namely the mechanisms (how) of the sponge, *T. hoshinota,* and its advantage (why) for killing corals. Two hypotheses were readily available to explain the mechanisms, the light blocking and the toxin hypotheses. It was noted that the two hypotheses were not mutually exclusive. Small patches of darkness and toxins were used in various in-situ experiments. The adaptive hypotheses for killing the corals were either to gain nutrients or to gain substrates.

## Results

### Experiment 1: Sponge fragments

Evidence of bleaching first occurred 3 days after the treatment and was only evident in the group with fragments of *T. hoshinota*. No bleaching was detected in the other 2 groups with the black cloth (to block light) and white cloth (control) (Table 1). Chi-square tests confirmed that the occurrence of bleaching depended on the treatments (*p* < 0.001 in both tests: sponge fragment vs. black cloth, and sponge fragment vs. white cloth).

**Table 1:** Coral responses in the sponge fragment test. *Terpios hoshinota* is more likely to cause coral bleaching (*T. hoshinota* vs. black cloth: *p* < 0.001; *T. hoshinota* vs. white cloth: *p* < 0.001, Chi-square tests).

Treatment/Coral response	Bleaching	Not affected
*Terpios hoshinota*	9	0
Black cloth	0	9
White cloth	0	7

### Experiment 2: Sponge mixture

**Figure 1:**
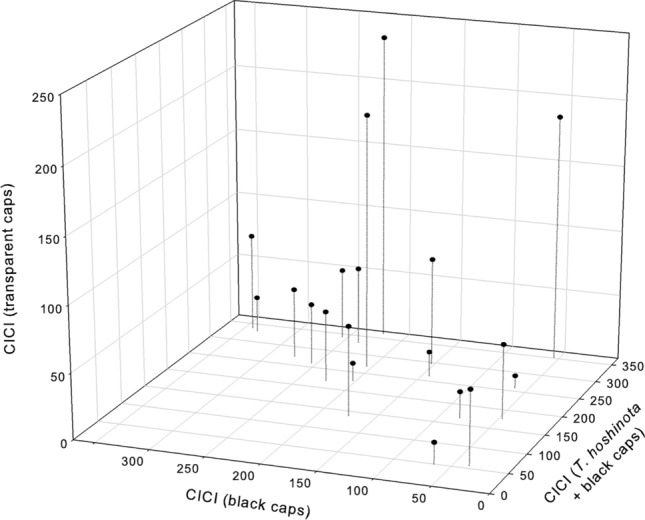
The color intensity change indices (CICI) of three treatments of the sponge juice experiment. CICI (S + B) > CICI (B) > CICI (T) (n = 19, *p* < 0.01 in all three pair-wise comparisons, Wilcoxon Signed Rank Tests). S: Sponge, B: Black caps, T: Transparent.

Significant difference in CICI was detected among the 3 treatments (*p* < 0.001; Friedman Test, n = 19; Fig. 1). The treatment with sponge and a black cap had the greatest effect, whereas the treatment with the transparent cap demonstrated the smallest response. All 3 pairwise comparisons were significant [CICI (sponge and black caps) > CICI (black caps) > CICI (transparent caps), n = 19, *p* < 0.01 in all 3 pair-wise comparisons, Wilcoxon signed-rank tests].

### Experiment 3: Sponge supernatant

**Figure 2:**
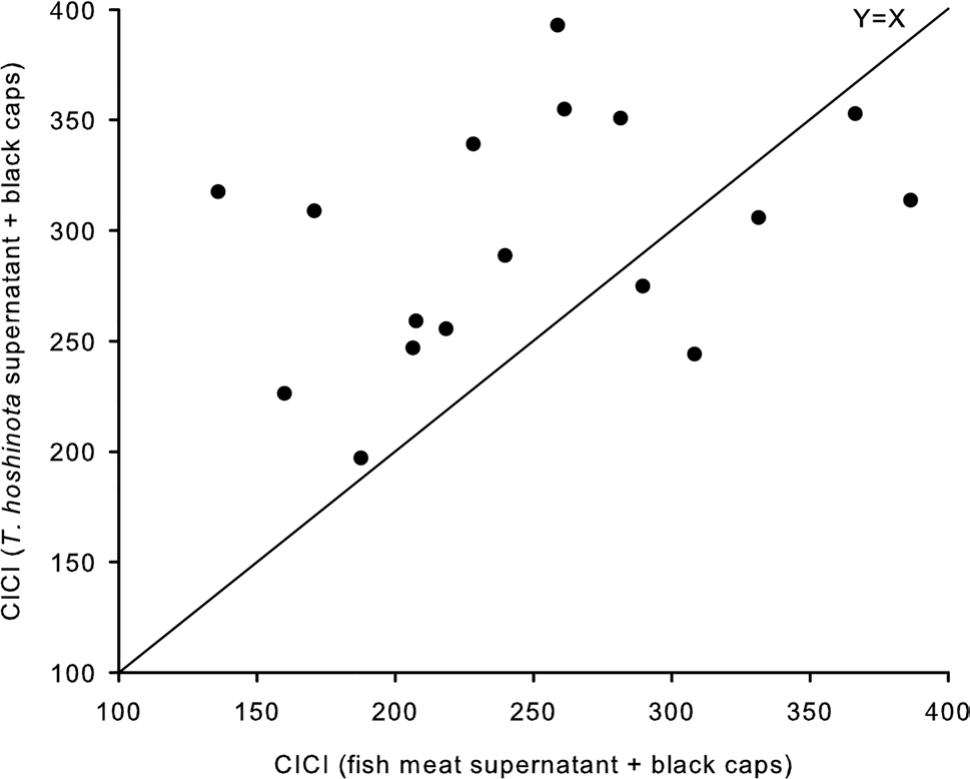
Comparison of color intensity change indices (CICI) between “F + B” and “S + B”. The line in the plot represents Y = X. CICI (S + B) > CICI (F + B) (n = 17, *p* = 0.028, Wilcoxon Signed Rank Test). F: Fish, B: Black caps, S: Sponge.

Two days after the treatment, a significant difference was detected between the sponge + black cap, and fish-meat + black cap groups (Fig. 2) with the former showing significantly greater effects.

**Figure 3:**
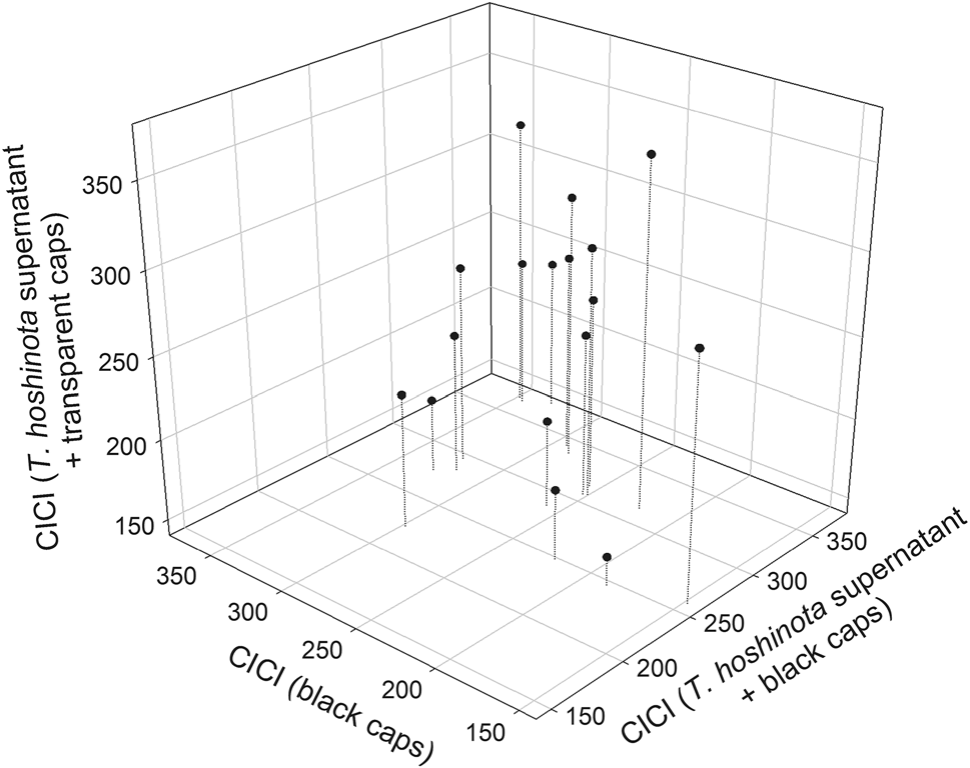
The color intensity change indices (CICI) of three treatments in sponge supernatant experiment. CICI (S + B) > CICI (B) = CICI (*S*) (n = 19, “S + B” vs. “B”: *p* = 0.055; “S + B” vs. “S”: *p* < 0.01; “B” vs. “S”: *p* = 0.12, Wilcoxon Signed Rank Test,). S: Sponge, B: Blackcaps.

Significant difference in CICI was observed among the 3 treatments, i.e., only the sponge supernatant, only black caps, and both sponge supernatant and black caps (n = 17; *p* < 0.01, Friedman Test, Fig. 3), 4 days after the treatment. Further pair-wise analyses indicated that both factors combined had stronger effects than when single factors were applied [*p* = 0.055 (against the black cap alone), *p* < 0.01 (against sponge alone), Wilcoxon signed-rank test, n = 17]. No significant difference was observed between the 2 single factors (*p* = 0.12; Wilcoxon signed-rank test, n = 17).

### Stable isotope experiment

Six pieces of transplanted corals, 3 from the isotope labeling group, and 3 from the control group, which had been covered by *T. hoshinota* were successfully retrieved. The other samples either lost their labels or were not covered by the sponge in the field.

The control corals had δ^13^C: − 14.4 ± 1.2‰, δ^15^N: 4.8 ± 0.6‰ (n = 3). The error terms were 95% confidence intervals of the means throughout this study. The sponges grown on the control corals had lower values, i.e., δ^13^C: − 21.4 ± 0.8‰, δ^15^N: 3.6 ± 0.1‰ (n = 3). The control corals had a significantly higher heavy stable isotopes content than the sponges grown on them (δ^13^C, *p* < 0.01; δ^15^N; *p* < 0.05; *t*-tests, n = 3, Table 2).

**Table 2:** The comparison of δ^13^C and δ^15^N between control corals and sponges grown on the control coral.

Stable isotope	Subject		
	Control corals	Sponges grown on the control corals	*p* value (*t*-tests)
δ^13^C (‰)	− 14.4 ± 1.2	− 21.4 ± 0.8	*p* < 0.01
δ^15^N (‰)	4.8 ± 0.6	3.6 ± 0.1	*p* < 0.05

**Figure 4:**
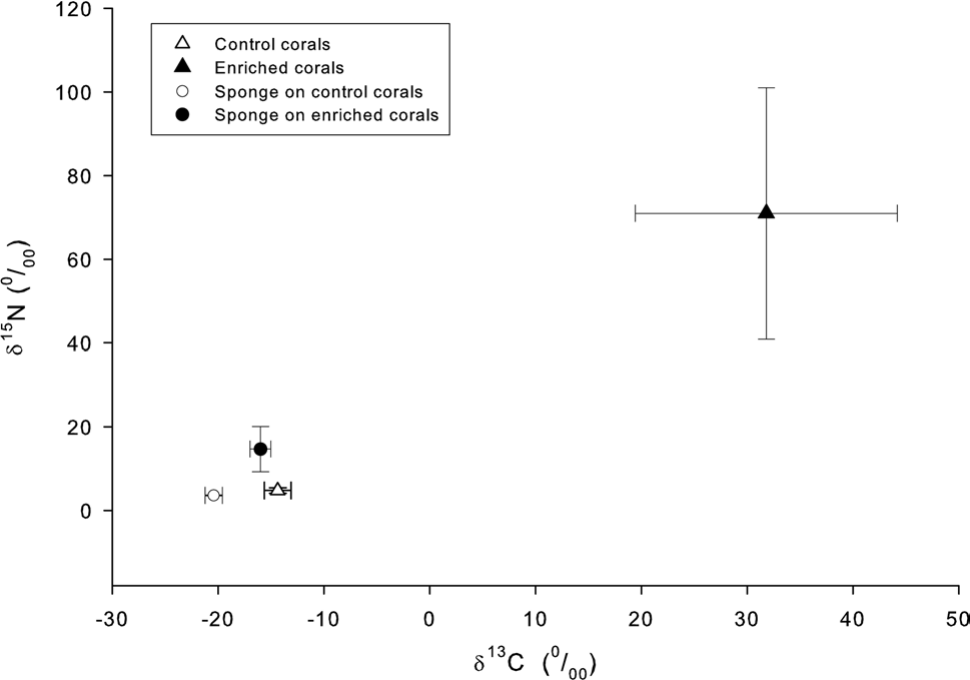
Stable isotope compositions (δ^13^C and δ^15^N) of control and artificially enriched corals and the sponge, *Terpios hoshinota,* covering the corals. Error bars indicate 95% c.i. Stable isotope composition of *T. hoshinota* covering enriched corals are significantly higher than those covering control corals (δ^13^C: n = 3, *p* < 0.01; δ^15^N: n = 3, *p* < 0.05, t-test,).

The enriched corals had δ^13^C: 31.8 ± 12.1‰, δ^15^N: 71.0 ± 29.5‰, and the *T. hoshinota* grown on them had lower values: δ^13^C: − 16.0 ± 1.0‰, δ^15^N: 14.7 ± 5.3‰ (n = 3). In fact, the stable isotope composition of *Terpios* on the enriched corals was closer to the isotope compositions of the sponges on the control corals, than to the coral tissues underneath them (Fig. 4).

**Figure 5:**
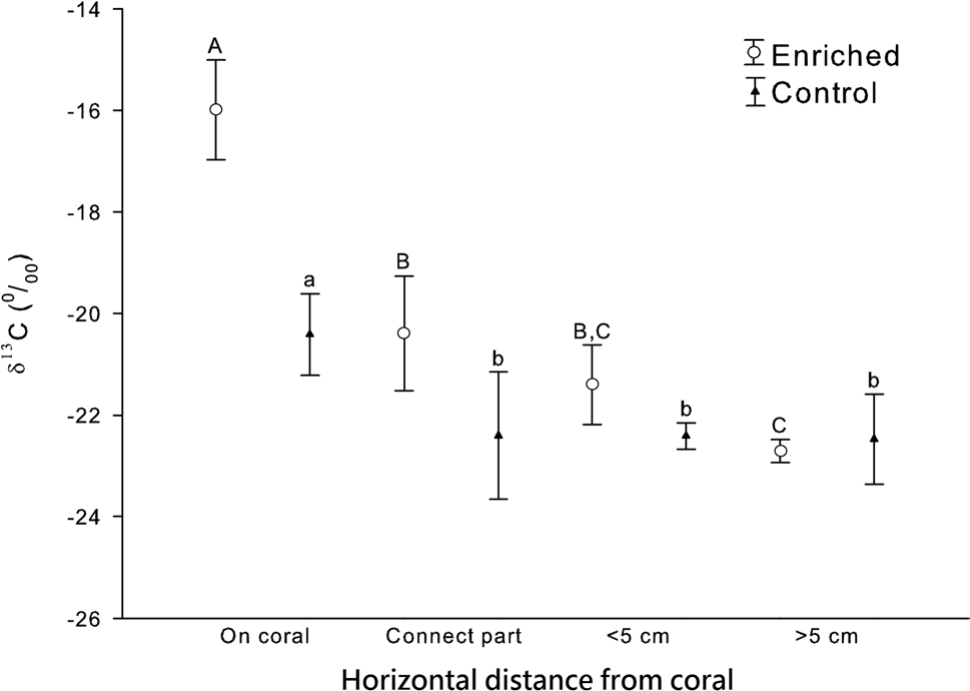
Stable carbon isotope composition (δ^13^C) of *Terpios hoshinota* grown on and at different positions in control and enriched (^13^C) corals. Error bars indicate 95% c.i. Different letters indicate significant difference (Upper letter: Enrich group; Lower letter: Control group).

Analysis of the sponges indicated that a significant difference in δ^13^C along the growth axis was evident only in the samples with the enriched treatment (ANOVA, F_2,6_ = 8.33; *p* < 0.05), but not in the sponges grown on the control corals (ANOVA, F_2,6_ = 0.01; *p* = 0.99; Fig. 5). A Fisher’s PLSD test indicated that the sponge δ^13^C was the highest directly on top of the enriched corals, intermediate at the junction, and the lowest at the > 5-cm positions (*p* < 0.01; n = 3), and the > 5-cm and the < 5-cm positions were not significantly different (*p* = 0.13; n = 3). In comparing the sponge δ^13^C between the enriched and control corals of the equivalent positions, a significant difference was only observed in the positions right above the transplanted corals (Fig. 5). This was a clear indication that the incorporated heavy stable C of the sponge was not translocated to the more proximal part of the sponges.

**Figure 6:**
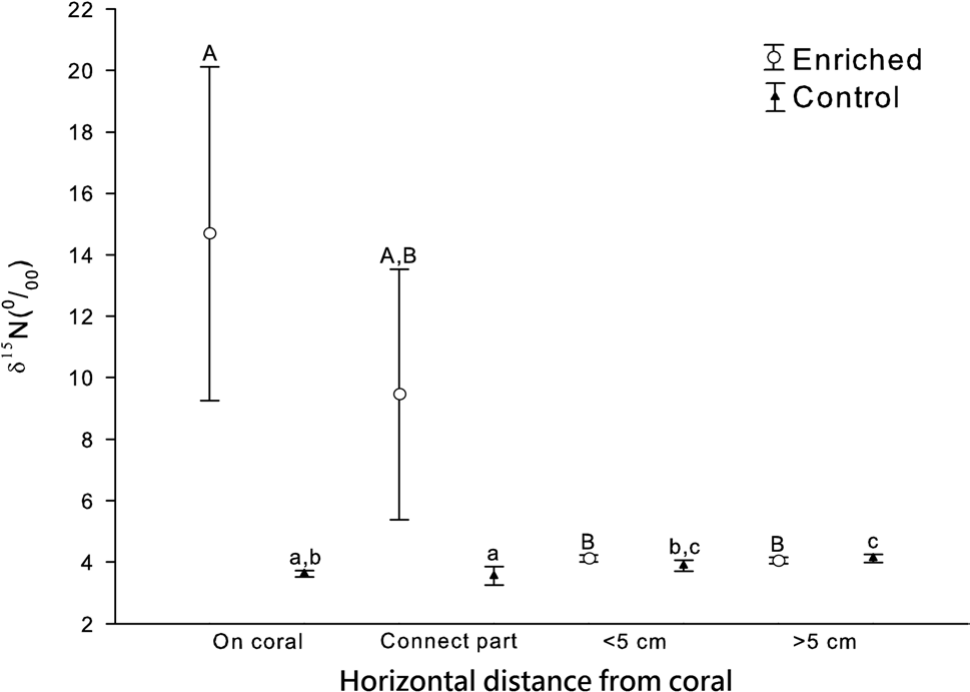
Stable nitrogen isotope composition δ^15^N of *Terpios hoshinota* grown on and at different positions in control and enriched (^15^N) corals. Error bars indicate 95% c.i. Different letters indicate the significant difference (Upper letter: Enrich group; Lower letter: Control group).

Concerning δ^15^N in the control group, significant difference was observed among the different positions of the sponge (ANOVA, F_2,6_ = 6.93; *p* < 0.05), although the difference was small. Lighter nitrogen was discovered near the expanding fronts (Fisher’s PLSD test, *p* = 0.01; n = 3; Fig. 6). In the enriched group, significant difference was observed among different positions of the sponge (ANOVA, F_2,6_ = 6.91; *p* < 0.01), but the trend was the opposite because the expanding fronts that covered the coral had higher levels of nitrogen. The junction was heavier than the more proximal part. By comparing the δ^15^N that grew on the 2 groups of corals, the sponges on the enriched corals had higher δ^15^N than those on the control corals in the newly grown tissues. However, the 2 groups of sponge tissues did not vary significantly in the more proximal parts that were far from the new growth (Figs. 5, 6). This result was consistent with the result of δ^13^C and did not support the suggestion that the frontal sponge tissues translocate acquired new materials to more proximate parts of the sponge.

### Estimation of underlying coral tissue contribution to sponge

Three possible sources contributed to the stable isotope compositions of the *T. hoshinota* sponge, namely the coral tissues underneath, microbes and food particles filtered from the water column, and the translocation of tissues or materials from the proximal part of the sponge. The stable isotope of the sponge could be expressed as the following equation:

1 $$\updelta _{{{\text{sponge}}}} =\updelta _{{{\text{coral}}}} \times f_{{{\text{coral}}}} +\updelta _{{{\text{water}}}} \times f_{{{\text{water}}}} +\updelta _{{\text{back sponge}}} \times f_{{\text{back sponge}}}$$

δ_sponge_, Isotope composition of new *T. hoshinota* tissues; δ_coral_, Isotope composition of coral tissues underneath *T. hoshinota*; δ_water_, Isotope composition of sponge food in the water column; δ_back sponge_, Isotope composition of the proximal part of *T. hoshinota*; *f*_coral_, Fraction contributed by underlying coral to new *T. hoshinota* tissues; *f*_water_, Fraction contributed by food particles in the water column to new *T. hoshinota* tissues; *f*_back sponge_, Fraction contributed by proximal part of the tissue to new *T. hoshinota* tissues.

$${\text{and}}\,f_{{{\text{coral}}}} + f_{{{\text{water}}}} + f_{{\text{back sponge}}} = \, 1$$

Assuming that δ_water_ × *f*_water_ + δ_back sponge_ × *f*_back sponge_ remained unchanged between treatments, the formula could be transformed to

2 $$\updelta _{{{\text{sponge}}}} = f_{{{\text{coral}}}} \times\updelta _{{{\text{coral}}}} + {\text{Constant}}$$

Therefore, the fraction of new sponge tissues contributed by the underlying coral tissues (*f*_coral_) could be estimated when the δ_sponge_ values under different δ_coral_ were available. Because the constant in Eq. (2) was not known, at least 2 pairs of data were required to estimate *f*_coral_. Using 3 paired samples of enriched coral tissues and the sponge tissues that covered them, the *f*_coral_ estimated from δ^13^C and δ^15^N were 9.5% and 16.9%, respectively, using regression. In Eq. (2), the enrichment of the heavy isotope composition along the food chain was not considered.

## Discussion

The sponge *T. hoshinota* killed stony corals effectively and it was accomplished by using both toxins and light-blocking. This conclusion was substantiated in Experiment 3, in which the combined effects of both factors were greater than individual factors (Fig. 3).

Secondary metabolites were used as protection against predators, or in competition with neighbors^22,26^. They were not only used as a defense mechanism, but as an aggressive tool against corals because the sponge clearly benefited from the presence of the stony corals^14^.

As a benefactor in an asymmetric (+ , −) interaction, *T. hoshinota* could be considered a predator, disease, or parasite, depending on how fast the corals were killed and whether most of the nutrients/energy was gained from the corals. Additionally, it was possible that *T. hoshinota* was targeting the substrate, rather than the nutrients/energy available in the tissues of stony corals. In the latter case of the substrate scenario, the nature of the interspecies relationship is similar to competition. However, in competition, both parties suffered (−, −) from the presence of the opponents, which was clearly not the case between *T. hoshinota* and its victims, the stony corals as revealed in this study. Similar interspecies relationships exist between epiphytes and their hosts, although epiphytes do not necessarily gain energy or nutrients from their hosts, and typically do not kill them^27^. This study demonstrated that *T. hoshinota* gained certain materials from the corals they covered. However, it was not clear whether the nutrient materials were acquired before or after coral decomposition. If latter, the intake of coral tissues may just be a side effect of coral mortality. Therefore, we did not make any adjustment in the equations (specify) based on the general patterns of heavy isotope enrichment along the food chainssee^28^. Thinesh et al.^28^ reported that the nutrient of *T. hoshinota* may come from the symbiotic cyanobacteria Thinesh et al.^29^.

Various sponges are known to have secondary metabolitese.g.,^30^ and many sponges are heavily pigmented, therefore light penetration through these sponge tissues must be low. *Terpios hoshinota* is unique because it is able to spread rapidly on live corals of shallow reefs^31^ and certain traits might enable such rapid expansion of the sponge. We suggest that the encrusting morphology couldplay an essential role. The relatively large surface area that is in contact with the coral tissues are very helpful, if *T. hoshinota* had adopted an erect or massive morphology, a 3D growth form would require much greater investment in biomass than if the sponge had been growing in 2D plane as at present. A 2D morphology is also efficient if chemical delivery and light-blocking prove to be weapons against underlying corals.

The reason *T. hoshinota* adopts an encrusting morphology may be adaptive in shallow waters. This is because the low-lying shape allows more resistance to wave actions and for sunlight reception by symbiotic cyanobacteria^11^ to access light. They could also play a critical role in defeating the corals that are known to have defense mechanisms against encroaching enemiese.g.,^32^. The pigments blocking light were likely contributed by the symbiotic cyanobacteria, because they were the most conspicuous cells in *T. hoshinota* tissues^24,33^. In contrast, the sponge cells were difficult to observe, and represented only a small fraction of the sponge holobiont in microscopic observations.

The stable isotope examinations in this study indicated that only a small proportion of *T. hoshinota* nutrients originated from the underlying corals. The first evidence was based on a comparison of the stable isotope compositions of non-enriched natural corals and the sponges covering them. If the corals had been a principle food source of *T. hoshinota*, the values of δ^13^C and δ^15^N of the sponge were expected to be higher than those of the corals because of the preferential metabolism of the lighter isotopes^28^. Second, when the sponge covered the enriched corals in our experimental setup, the new sponge tissues had substantially lower heavy isotope signals than the underlying corals did. This finding provided additional support for the suggestion that the underlying corals were not the principal nutrient source of the sponges, and that the nutrient hypothesis was therefore not well supported. According to our calculations, the underlying coral contributed approximately 10% of the carbon and 17% of the nitrogen to the encrusting sponges. Theoretically, these values could be an underestimation of the total input from the underlying corals if the new sponge tissues that covered the corals transported materials to more proximal parts of the sponges. We assessed this possibility by sampling various parts of the experimental sponges, and sponge tissues up to 8 cm horizontal distance off the underneath corals were among the samples analyzed. However, comparisons within and between the sponges growing on treatment (enriched) and control corals revealed no evidence of translocation in either N or C (Figs. 5, 6).

In contrast, other sources of nutrients could have originated from particles in the water column, from other parts of the sponges, or from symbiotic cyanobacteria. The experimental design of this study was not able to distinguish these sources.

*Terpios hoshinota* contains high concentrations of cyanobacteria^24,33^, therefore, the organic carbon may have originated from inorganic forms first incorporated by the photosynthetic symbionts of the sponge (based on unpublished stable isotope studies on the incorporation of inorganic carbon into cyanobacteria; personal communication with YL Lee, National Sun Yat-sen University). The latter carbon source may contribute substantially to the carbon pool of the sponge and lowers the relative contribution from the underlying coral tissues, compared with nitrogen (10% vs. 17%). Alternatively, the potential retention and recycling of nitrogen within the sponge holobiont did not cause the difference in the relative contribution of C and N from the underlying corals. This is because the nutrient recycling is not source sensitive. In addition, filter feeding could potentially contribute significantly to this low-lying sponge as has been shown in an ink experiment (http://www.youtube.com/watch?v=ZxqAzY_T1q8), which demonstrated that *T. hoshinota* effectively pumped surrounding water through their tissues.

*Terpios hoshinota* clearly did not rely on underlying corals as a major source of nutrients. We suggest that coral nutrients assist, but are not critical to, sponge expansion. The observation that *T. hoshinota* could be grown on glass slides, plastic sheets, and rubber tires^16^ further undermines the nutrient hypothesis, but is compatible with the substrate hypothesis. Live corals possess and generate precious resources, such as substrate, that *T. hoshinota* has evolved to exploit. Therefore, a reef with high coral coverage provides ample opportunities for the black sponge, and the nutrients available in the coral tissues were not the primary target of the black sponge, but a bonus.

## Materials and methods

### How the sponges kill the corals

**Figure 7:**
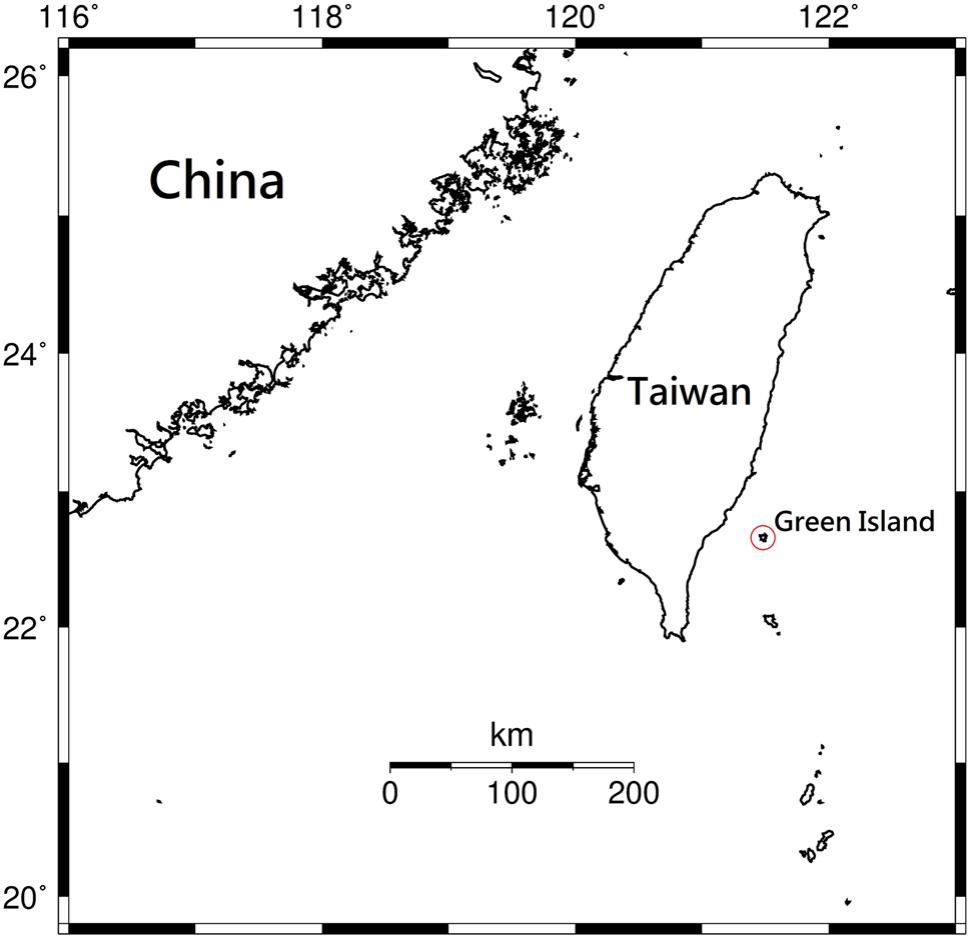
The study site, Green Island, in this research. The figure was created by Generic Mapping Tools (GMT version 5.4.2)^34^.

The sponge samples used in this investigation were all collected on Green Island (22° 40′ N, 121° 29′ E), southeast of Taiwan (Fig. 7).

Three experiments, with different levels of sponge modifications were used to test the toxin and the light blocking hypotheses. In the first experiment, fresh sponge fragments were used, and in the second, a mashed mixture of sponge tissues was used. In the third experiment, only the supernatant of raw extracts after centrifugation were used. Phytagel (Sigma P8169, CAS 71010-52-1) was used as a medium because of its ability to release contents slowly^35^. The magnitude of color change of the coral tissues was used as an index of the strength of toxicity and is discussed in detail in the image analysis section.

### Experiment 1: Sponge fragments

**Figure 8:**
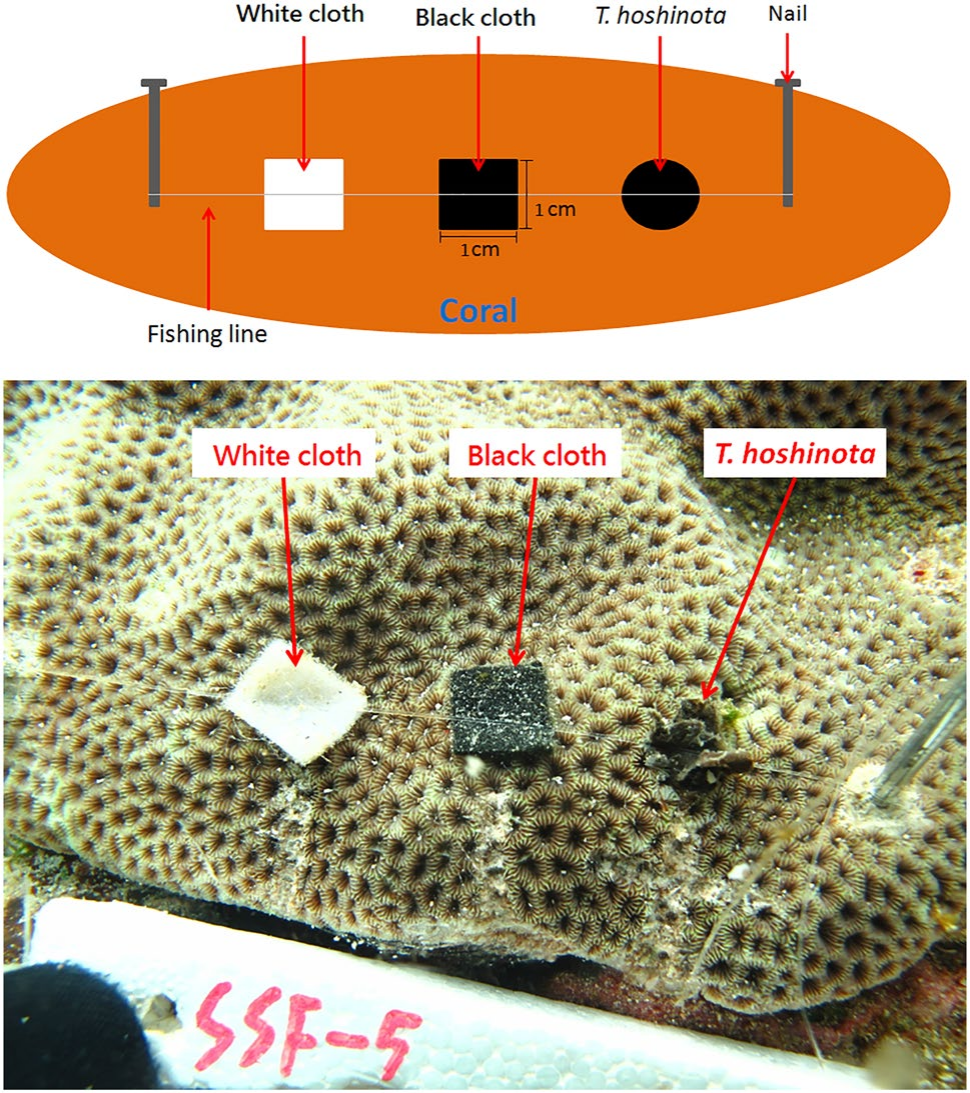
The design of sponge fragment experiment.

To test the toxin hypothesis, 3 treatments of sponge fragments were used (black cloth for light blocking hypothesis, and white cloth for control were used to cover parts of the same coral colonies in a paired-design experiment). Sponge fragments were collected from the margins of *T. hoshinota* by scuba divers and were trimmed to approximately 1 × 1 cm sheets. They were then each fixed on the same coral colonies by using fish lines and nails accompanied by black and white cloth of the same size (Fig. 8). A total of 9 coral colonies were used in this experiment. The set up was initiated on 9 February 2012, and 3 days later, the responses of the coral tissues underneath the cloth were checked and photographed for signs of bleaching.

### Experiment 2: Sponge mixture

Fresh sponge samples of *T. hoshinota* were cleaned of other organisms and sand grains before being blotted dry and their volume was estimated using a water displacement method. To test the toxin and light blocking hypothesis, the sponge mixture was prepared using 24 mL of tissues that were added to a solution containing 1.4 g of Phytagel and 216 mL of seawater, and subsequently thoroughly mixed before they were cured in black caps. Black and transparent caps with only Phytagel were used to test the light blocking hypothesis without sponge tissues. These caps with gels were preserved in a refrigerator before being used within 24 h.

**Figure 9:**
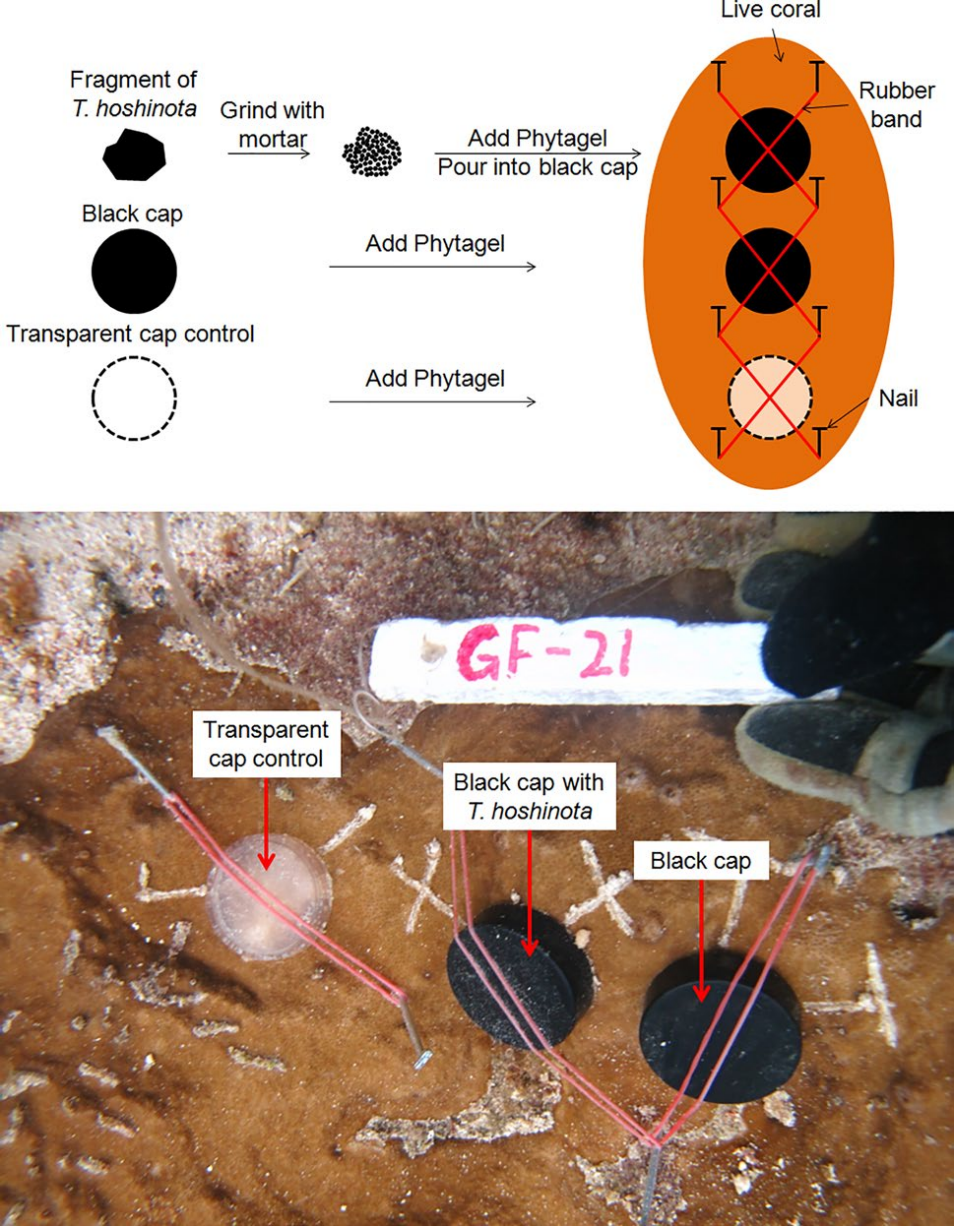
The design of sponge mixture experiment.

A total of 19 replications were used, each containing one black cap with sponge mixture, one black cap without sponge mixture, and one transparent cap without sponge mixture. Each replicate of 3 caps (treatments) were used on one coral colony and were attached using rubber bands and nails (Fig. 9). An underwater camera (Canon G10) was calibrated to measure the color temperature onsite before photographing each replicate of the 3 caps. The camera used the aperture priority (f = 2.8) automatic exposure without flash. This experiment started on 26 February 2012, and the results were checked and photographed 3 days later.

### Experiment 3: Sponge supernatant

The sponge tissues were cleaned before being homogenized for 5 min. Next, from 44.5 g wet weight of sponge, 22.67 mL of clear suspension was obtained after centrifuging the sample (Sigma 2-16P) at 4000 rpm for 10 min. As a control, the same procedure was conducted on 4.32 g of fish meat (yellow fin tuna) and 35 mL of clear supernatant was obtained. The protein contents of both were measured. The same final concentration, 87.5 mg ml^−1^ of proteins, was used in Phytagel.

**Figure 10:**
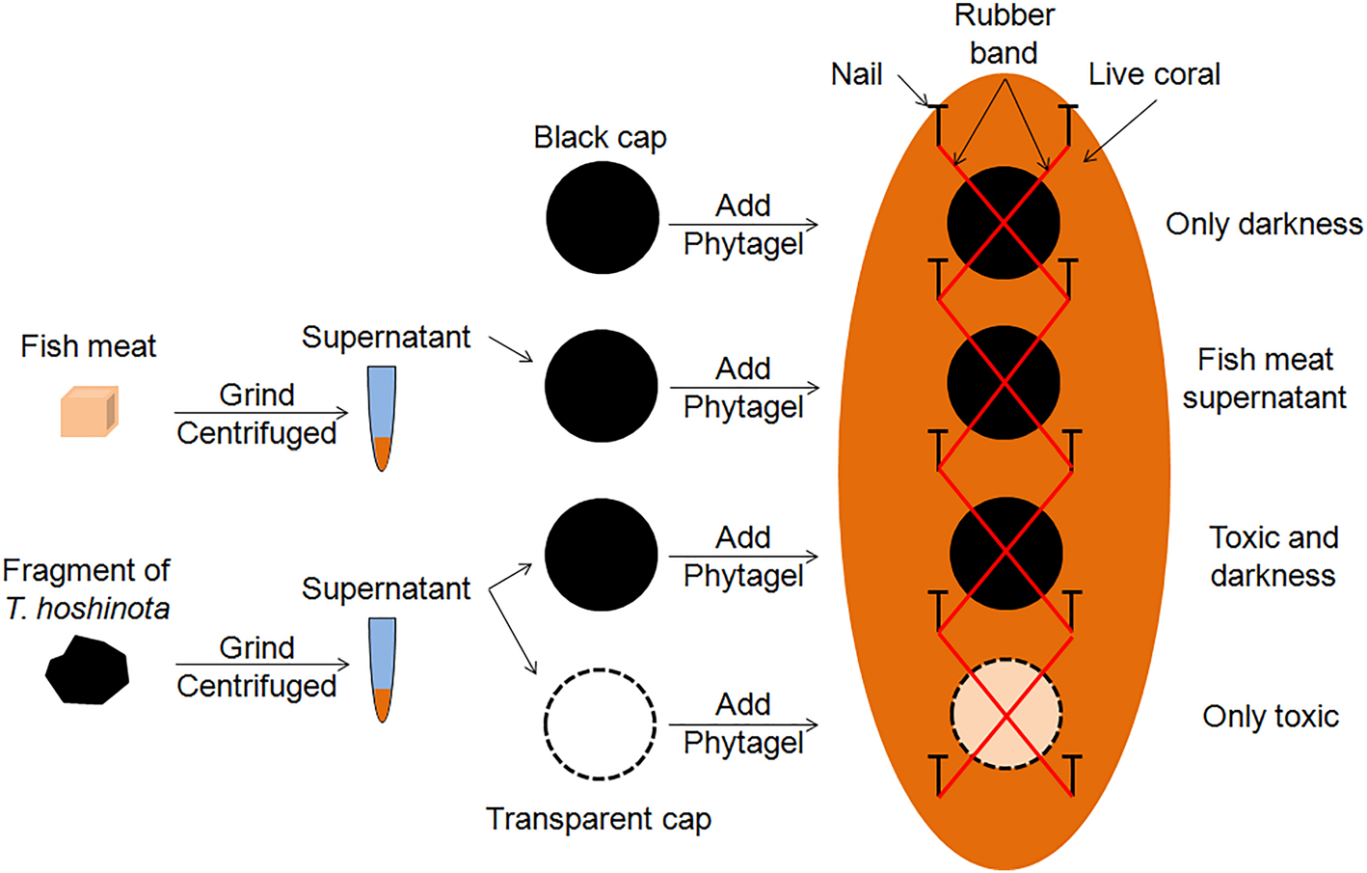
The design of sponge supernatant experiment.

Four treatments were involved, namely, the clear sponge supernatant in black caps, clear sponge supernatant in transparent caps, and a blank sample of only Phytagel in black caps (Fig. 10), in addition, a fish meat control was used to control for the possible effect of sponge protein decomposing on coral polyps. A total of 17 replications, each on a colony of massive *Porites* spp. was used. Additional procedures followed are described in Experiment 2. The experiment started on 10 August 2012, and the responses of the coral tissues were checked and photographed on 14 August 2012.

### Image analysis

Ten 50 × 50 pixel areas were selected from each of the photographs taken below the caps, which covered and were in contact with parts of the corals. The surface of the *Porites* colonies was not smooth, and only the elevated parts, such as the common walls between corallites were selected for comparison because they were most likely to contact the gel. The average light intensities of red, green, and blue were obtained using ImageJ for each area. The color intensity change index (CICI) between the treated and control areas in the same pictures could then be calculated by summing the absolute differences among all 3 colors. This method was applied to Experiments 2 and 3.

### Statistics

In each experiment, the CICI was compared among the 3 treatments, using Friedman tests. Additional pair-wise comparisons were performed using Wilcoxon signed rank tests if significant differences were observed in the Friedman test results.

### Stable isotope experiment

Natural stable isotope compositions, as well as heavy isotope enriched corals were used to assay whether and how much of the sponge composition originated from the underlying corals.

Twelve pieces of massive *Porites* spp. (each approximately 10 × 5 × 5 cm) were collected and 6 were immersed in water with additional ^13^C and ^15^N [NaH^13^CO_3_, (Sigma-Aldrich, 98 atom %) at 10 μM and Na^15^NO_3_ (Europa Scientific, 99.8 atom %) at 0.2 μM]. The other 6 pieces were immersed in control seawater, which did not contain additional stable isotopes. Both treatments were placed in a water bath and were oscillated at 1 Hz outdoor under the afternoon sun for 2 h. The water bath was maintained at room temperature by using flowing tap water. After immersion, each coral was separated into 2 pieces. The first was processed to collect coral tissues, and the second was fixed next to a large *T. hoshinota* sponge at 2–3 m depth at Gongguan, on the northern coast of Green Island. The sponge grew over the coral pieces over a period of approximately one month and was subsequently retrieved. The coral and sponge samples were stored below − 20 °C until processing.

The coral samples after the sponge coverage were decalcified in a 1:1 mixture of 50% HCOOH and 20% CH_3_COOH. The decalcified tissues were rinsed twice in deionized water. Two pieces of 1 × 1-cm tissues were collected from each sample, and the sponge tissues were separated from the underneath coral tissues, while other organisms in the tissues were subsequently removed. The cleaned tissues were then immersed in 0.1 N phosphoric acid for 1 d to dissolve any residual CaCO_3_. They were rinsed 3 times in deionized water before being oven dried at 60 °C for 7 days. For each sample, approximately 1 mg of dry weight was placed in tin capsules for stable isotope analysis, which was conducted by the Stable Isotope Facility of University of California at Davis. The instrument used was a PDZ Europa ANCA-GSL elemental analyzer and PDZ Europa 20–20 isotope ratio mass spectrometer (Sercon Ltd., Cheshire, UK). The standard errors of δ^13^C and δ^15^N were 0.2‰ and 0.3‰, respectively.

**Figure 11:**
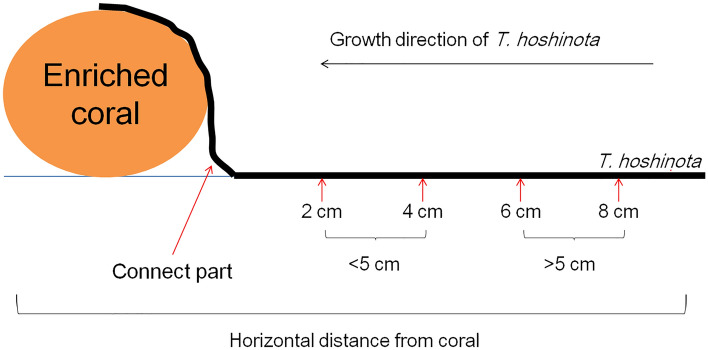
Sponge sampling positions in the nutrient transport experiment.

To test if nutrients of the expanding ends were translocated to the proximal parts of the sponges, 5 additional sponge tissues were serially collected at 2-cm intervals in the area at the back of coral (the proximal parts). The first sample was located at the junction of the original substrate of *T. hoshinota* and the transplanted corals, and the last was 8 cm from the first. The sample preparation followed the same procedures mentioned previously. Certain sample sizes did not reach the minimal amount required (0.2 mg of dry weight) for the stable isotope analysis. Therefore, the 2-cm and 4-cm samples were combined to form a new group: < 5 cm, and the 6-cm and 8-cm samples were combined to form the > 5-cm group (Fig. 11).
